# CircPWWP2A promotes renal interstitial fibrosis through modulating miR-182/ROCK1 axis

**DOI:** 10.1080/0886022X.2024.2396455

**Published:** 2024-09-04

**Authors:** Qian Huang, Kaiyi Zhong, Jiali Wei

**Affiliations:** a Department of Nephrology, Haikou Third People’s Hospital, Haikou, Hainan, China; b Department of Nephrology, Danzhou West Central Hospital, Danzhou, Hainan, China; c Department of Nephrology, Hainan General Hospital (Hainan Affiliated Hospital of Hainan Medical College), Haikou, China

**Keywords:** Renal fibrosis, CircPWWP2A, miR-182, ROCK1, mitochondrial dysfunction

## Abstract

Renal fibrosis is a long-term and progressively worsening condition that impacts kidney function during aging and in the context of chronic kidney disease (CKD). CKD and renal fibrosis affect approximately 10% of the global population and are prevalent in about half of individuals over the age of 70. Despite ongoing research, the mechanisms underlying renal fibrosis are still not well understood, and there is currently a lack of effective treatments available. In the present study, we demonstrated a significant increase of circPWWP2A in renal tubular cells both in vivo and in vitro models of renal fibrosis. Suppressing circPWWP2A has the potential to reduce mitochondrial dysfunction and the production of mitochondrial reactive oxygen species (mtROS), ultimately leading to the inhibition of renal fibrosis. Whereas, supplementation of circPWWP2A led to more serve mitochondrial dysfunction, mtROS production and renal fibrosis. Mechanistically, we found the expression of circPWWP2A was negatively correlated with the expression of miR-182. And we further confirmed miR-182 was the direct target of circPWWP2A by dual-luciferase reporter assay and RIP assay. Then, we found miR-182 suppressed the expression of ROCK1 in both in vitro and in vivo models of renal fibrosis. Luciferase microRNA target reporter assay further indicated ROCK1 as a direct target of miR-182. Knockdown of ROCK1 inhibits renal fibrosis and mitochondrial dysfunction, suggesting ROCK1 not only served as an injurious role in mitochondrial homeostasis but also a pro-fibrotic factor in CKD. Taking together, our findings suggest that circPWWP2A may promote renal interstitial fibrosis by modulating miR-182/ROCK1-mediated mitochondrial dysfunction.

## Graphic Abstract

**Figure:**
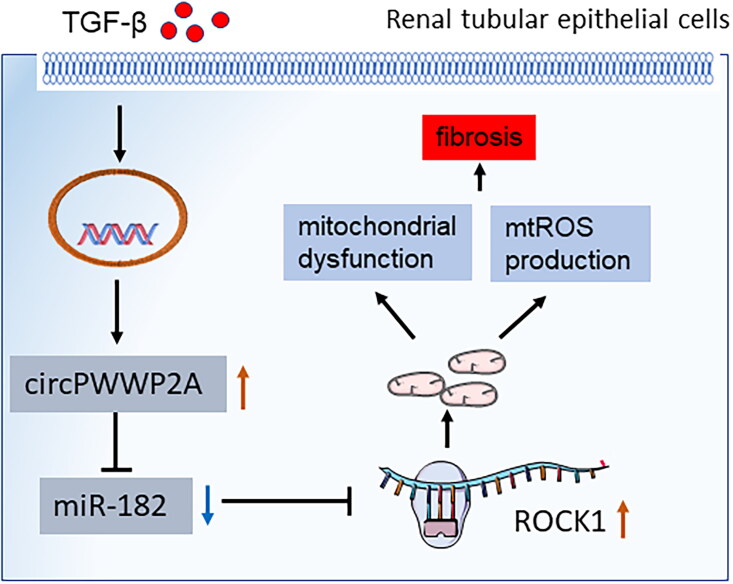

## Introduction

Chronic kidney disease (CKD) has a high incidence and low awareness rate, with approximately 10.5% to 13.1% of the population worldwide suffering from CKD [1,2]. Because of the hidden onset of CKD, it is often easy to be ignored in the early stage. If it is not found in time and active and effective treatment measures are taken, it will eventually progress to end-stage kidney disease (ESRD) [3]. At present, there is no specific drug treatment for ESRD, and only dialysis or kidney replacement treatment can be relied on, which greatly shortens the survival period and brings a huge burden to the family and society. The ultimate pathological outcome of numerous CKD is renal fibrosis. This condition is indicative of the kidney’s unsuccessful attempt to heal after prolonged and persistent injury, characterized by tubular atrophy, glomerulosclerosis, and interstitial fibrosis [3]. Thus, it is important to investigate the molecular mechanism of renal fibrosis and find the key molecular targets for delaying the progression of CKD.

Renal fibrosis is often accompanied by characteristic pathological changes such as inflammatory response, reactive oxygen species (ROS) production, epithelial mesenchymal transformation (EMT) and Extracellular matrix (ECM) deposition [1,2,4]. In CKD, renal tubular epithelial cells release pro-fibrotic cytokines, such as transforming growth factor beta (TGF-β), fibronectin (FN) and collagen I, which lead to ECM deposition and renal fibrosis. Upon which, TGF-β, as the most important pro-fibrotic factor, is the main driving force of renal fibrosis [5]. In addition, excessive ROS production in CKD will lead to the occurrence of oxidative stress in the body, and mitochondria is the most important part of the generation of ROS in the cell, and also the primary target organelle of oxidative stress damage. Thus, mitochondrial oxidative damage and dysfunction are associated with the development of CKD and renal fibrosis [6]. Kidney is a high energy metabolic organ with abundant mitochondria, and mitochondrial dysfunction may play a key role in CKD, which has attracted more and more attention in recent years [7]. Enhancing the mitochondrial antioxidant defense by using mitochondria-targeted antioxidants like MitoQ or SS-31 has demonstrated the ability to alleviate mitochondrial dysfunction and reduce kidney damage in animal models of diabetic kidney disease (DKD) and CKD induced by unilateral ureteral obstruction (UUO) [7–10]. Under normal conditions, mitochondrial homeostasis is regulated by mitochondrial division and fusion proteins.

Dynamin-related protein 1 (Drp1) plays a crucial role in the process of mitochondrial fission, where it relocates to the division site on the outer mitochondrial membrane to facilitate this process. On the other hand, Mitofusin-1 (Mfn-1), Mitofusin-2 (Mfn-2), and Opa-1 are integral proteins situated in the outer and inner membranes of mitochondria, and they work together to regulate mitochondrial fusion [11]. In research carried out by Sun et al. findings revealed that the expression of the mitochondrial fission protein Drp1 was elevated, while the levels of mitochondrial fusion-related proteins Opa1 and Mfn2 were reduced in both in vivo and in vitro models of renal fibrosis. Importantly, reversing these changes could alleviate mitochondrial dysfunction, finally playing the role of kidney protection [12]. Thus, it is suggested that mitochondrial dysfunction plays a key role in the renal fibrosis. However, the specific upstream molecular regulatory mechanism of mitochondrial dysfunction in renal fibrosis is rarely reported and needs further study.

In recent years, circular RNAs (circRNAs) have garnered substantial attention from the scientific community. These RNA molecules are a distinctive class of non-coding RNAs characterized by their unique back-splicing mechanism, which results in a covalently closed loop structure. This particular formation of circRNAs distinguishes them from linear RNAs and suggests potential functional roles in various biological processes and disease states [13]. Growing research has established that circRNAs can modulate gene expression either at the transcriptional stage or through post-transcriptional mechanisms. This regulatory effect is achieved through their interactions with microRNAs (miRNAs) or other molecular entities, highlighting their role in the complex network of gene regulation [14]. Increasingly evidences suggest that circRNA is involved in the pathogenesis of certain kidney diseases, and can be involved in the regulation of programmed cell death, invasion and metastasis, and even acts as a biomarker for certain kidney disease [15]. In renal fibrosis, Wen et al. indicated that circACTR2 could promote renal fibrosis by promoting IL-1β, Fibronectin, and collagen production in tubular epithelial cells [16]. In another study, Ge et al. showed that circ_0000064 could aggravate renal fibrosis in rats by regulating miR-243 [17]. Thus, circRNA may be a key regulatory factor in renal fibrosis, which is worthy of further study. Hsa_circ_0074837 is derived from the PWWP2A gene, and its splicing maturity sequence length is 965 bp, so it is also called circPWWP2A. It has been reported that TGF-β1 could promote the expression of circPWWP2A, thus leading to liver fibrosis, and knockdown circPWWP2A can inhibit the process of liver fibrosis in mice, indicating circPWWP2A is involved in the pathogenesis of fibrosis [18]. Based on this study, we believe circPWWP2A may also could promote renal fibrosis and it also has the potential to serve as a new therapeutic target.

In this study, we demonstrated a significant increase of circPWWP2A in renal tubular cell both in vivo and in vitro models of renal fibrosis. Functionally, inhibition of circPWWP2A attenuated renal fibrosis, whereas supplementation of circPWWP2A led to more serve renal fibrosis. Interestingly, we have further identified that circPWWP2A promoted renal interstitial fibrosis mainly through modulating miR-182/ROCK1 axis. This finding may provide a potential therapeutic target for renal fibrosis.

## Materials and methods

### Ethics statement

This research received approval from the Laboratory Animal Ethics Committee of Hainan General Hospital and was conducted in strict adherence to the institution’s guidelines. All measures were taken to ensure the welfare of the animals and to minimize any potential suffering throughout the study.

### Chemicals and reagents

The antibodies utilized in this study were sourced from various suppliers: Anti-Fibronectin (ab2413), anti-α-SMA (ab5694), anti-Drp1 (ab184247), anti-Mfn1 (ab221661), and anti-Opa1 (ab157457) were obtained from Abcam (Cambridge, UK). Anti-GAPDH (5174) was acquired from Cell Signaling Technology (Boston, MA). For immunoblotting, all secondary antibodies were purchased from Thermo Fisher Scientific (Waltham, MA). Additionally, FITC-conjugated goat anti-rabbit IgG was sourced from Abcam. miR-182 mimic and ROCK-1 siRNA were from Ruibo (Guangzhou, China). Fasudil was from Selleck Chemicals (Hoston USA).

### Animals and UUO induction

Male C57BL/6 mice, aged 8 weeks, were acquired from the Slaccas Animal Laboratory located in Haikou, China. These mice were maintained in a controlled environment with a stable temperature of 22 °C and a 12-h period of darkness. The experimental procedures were carried out following the approval and guidelines set forth by the Institutional Animal Care and Use Committee. In summary, the mice were sedated using pentobarbital at a dosage of 60 mg/kg. To ensure their body temperature remained around 36.5 °C, a rectal probe connected to a Homeothermic Blanket Control Unit (507220 F, Harvard Apparatus) was used. A minor surgical incision was performed on the left flank to access and isolate the ureter. Two ligatures were placed in the middle segment of the left ureter using 4–0 silk thread. In contrast, control mice underwent the same procedure to expose the left ureter but without performing any ligation. Fasudil, a well-known ROCK1 inhibitor administered at a dose of 60 mg/kg, was injected intraperitoneally every day starting on the day of the UUO surgery and continued until the mice were sacrificed. Additionally, in select experiments, miR-182 mimics (3 mg/kg) or control oligonucleotide LNAs were injected daily into the tail vein following the UUO procedure. The mice were euthanized seven days post-surgery, and the obstructed kidneys were harvested for subsequent morphological and biochemical assessments [19].

### Cells and TGF‐β treatment

The Boston University mouse proximal tubular cell line (BUMPT) was grown in DMEM medium supplemented with 10% fetal bovine serum. To prepare for experimentation, BUMPT cells were seeded at a density of 0.3 × 10^6 cells per 35 mm dish and were allowed to starve overnight in serum-free DMEM. Following this, the cells were treated with 5 ng/mL TGF-β for a duration of 48 h, while control cells were kept in serum-free DMEM without TGF-β. In certain experiments, 200 nM of microRNA mimics, 40 nM ROCK1 siRNA, or control oligonucleotides were introduced into the BUMPT cells using Lipofectamine 2000 according to the manufacturer’s protocol. Each in vitro assay was performed a minimum of three times to ensure reliability [19].

### Histological analysis of kidney tissues

For histological analysis, kidney tissues were initially preserved in a 4% paraformaldehyde solution to maintain their structural integrity. After fixation, the samples were embedded in paraffin and cut into 4 μm thick sections. The sections underwent staining using Hematoxylin and Eosin (H&E) to highlight general tissue morphology and Masson’s trichrome stain to specifically assess fibrosis. These staining procedures followed established protocols provided by Servicebio to ensure accurate and consistent results.

### Transmission electron microscopy

For electron microscopy analysis, kidney tissues were collected from both renal UUO mice and sham-operated control mice. The samples were initially fixed using a mixture of paraformaldehyde and glutaraldehyde (Sigma–Aldrich, 340855) to preserve cellular structures. Following fixation, the tissues were post-fixed with osmium tetroxide (Sigma–Aldrich, 201030), dehydrated through a series of ethanol solutions, and then embedded in Epon resin (Sigma–Aldrich, 45345). Once the Epon was polymerized, tissue blocks were cut into 70-nm-thick sections using a microtome (Leica, Germany). These sections were stained sequentially with uranyl acetate (TED PELLA, 19481) and lead citrate (Sigma–Aldrich, 15326) to enhance contrast. The stained sections were first examined at a lower magnification (×3,000) to identify representative proximal tubules, and then observed at higher magnification (×10,000) to closely analyze the mitochondria within these cells.

### Determination of reactive oxygen species

To measure mitochondrial reactive oxygen species (ROS) levels in BUMPT cells, MitoSOX Red (Thermo Fisher Scientific, M36008) was used according to the manufacturer’s protocol. Initially, cells cultured on coverslips were thoroughly washed twice with phosphate-buffered saline (PBS) to remove any remaining medium. The cells were then incubated with 5 μM MitoSOX Red at 37 °C for a duration of 10 min, allowing the dye to selectively enter the mitochondria and react with ROS. Following the incubation period, the coverslips were subjected to three additional washes with PBS to eliminate any unbound dye. This process ensured that the measured ROS levels accurately reflected mitochondrial activity.

### Fluorescence in situ hybridization (FISH)

For the FISH assay, probes were specifically designed and synthesized: one probe labeled with Fluor 488 to target circPWWP2A and another labeled with Alexa Fluor 555 for miR-182 detection. After fixing the cells to preserve their structure, they were incubated with a prehybridization buffer to prepare for the hybridization process. The hybridization step was carried out at 55 °C for 2 h, allowing the probes to selectively bind to their complementary RNA targets within the cells. Following hybridization, the cell nuclei were stained with DAPI, a fluorescent dye that binds to DNA, providing a clear contrast against the probe signals. The detection and analysis of the probe signals were conducted using the FISH Kit from RiboBio (Guangzhou, China), following the detailed instructions provided by the manufacturer to ensure precise and reliable results.

### Immunoblot analysis

Total protein extraction from renal tissues or cultured cells was carried out using a radioimmunoprecipitation assay (RIPA) lysis buffer, which included protease inhibitors to safeguard against protein degradation. The proteins were then subjected to separation via 12% sodium dodecyl sulfate polyacrylamide gel electrophoresis (SDS-PAGE), which enabled their fractionation based on molecular size. Following this separation, the proteins were transferred to a polyvinylidene fluoride (PVDF) membrane for further analysis. The membrane was first incubated with a blocking solution of 5% skim milk to prevent nonspecific binding of antibodies. Subsequently, it was probed with specific primary antibodies, followed by an incubation with a horseradish peroxidase (HRP)-conjugated secondary antibody to enable detection. The protein bands on the membrane were then visualized using an enhanced chemiluminescence (ECL) detection system (Amersham Pharmacia Biotech, Little Chalfont, UK), providing high sensitivity and allowing for accurate quantification of protein expression levels.

### Immunofluorescence

Kidney sections embedded in paraffin were processed through a series of steps including deparaffinization, rehydration, and antigen retrieval. For antigen retrieval, the sections were incubated in a 0.1 M sodium citrate buffer (pH 6.0) at 100 °C. Following this, the tissue sections or fixed cells were permeabilized using 0.1% Triton X-100 and subsequently treated with a blocking buffer to reduce nonspecific binding. The specimens were then exposed to specific primary antibodies, incubated overnight at 4 °C. After primary antibody incubation, the sections were treated with either FITC- or rhodamine-conjugated secondary antibodies for 1 h at room temperature, followed by staining with DAPI (MilliporeSigma, D9542) to highlight the nuclei. Finally, the slides were analyzed under a fluorescence microscope, and representative images were captured and displayed to illustrate the findings.

### qPCR

Total RNA was extracted from kidney tissues or cultured cells using TRIzol reagent (Invitrogen; MA), which effectively isolates RNA by disrupting cellular components. Following extraction, the RNA from each sample was converted into complementary DNA (cDNA) using the M-MLV Reverse Transcriptase cDNA Synthesis Kit (TaKaRa Bio USA). Quantitative PCR (qPCR) was then conducted with the SYBR Premix Ex Taq^™^ II (TaKaRa Bio USA), which provides precise measurement of gene expression levels. The PCR results were analyzed using the LightCycler 96 SW 1.1 software to generate quantitative data. The expression levels of the genes of interest in each sample were calculated and presented as 2^(−ΔΔCt) values, reflecting relative changes in gene expression. This approach ensured accurate quantification and comparison of gene expression across different samples.

### Dual-luciferase reporter assay

HEK-293T cells were cultured and plated into 24-well tissue culture plates to prepare for subsequent transfections. The pmirGLO reporter vector, obtained from Promega, which includes either the wild-type (WT) or mutant-type (MUT) constructs of circPWWP2A and ROCK1, was introduced into the HEK-293T cells. This transfection was carried out in conjunction with either miR-182 mimics or negative control (NC) mimics. The transfection process utilized Lipofectamine 2000 reagent (Invitrogen, USA), which facilitates the efficient delivery of the plasmid DNA and RNA mimics into the cells. This experimental setup aimed to evaluate the interactions between circPWWP2A, ROCK1, and miR-182 in the context of gene regulation and reporter gene expression.

### RNA immunoprecipitation (RIP) assay

For the RNA immunoprecipitation (RIP) assay, the Magna RIP kit (Millipore, Billerica, MA, USA) was utilized to capture RNA-protein complexes. In this procedure, cell lysates were mixed with RIP buffer containing magnetic beads that were either conjugated with anti-Ago2 antibody (Millipore, Billerica, MA, USA) or with a control antibody (IgG). After binding, the beads were subjected to multiple washes with a wash buffer to remove nonspecific interactions. To purify the RNA, the complexes were then treated with 0.1% SDS and proteinase K to digest proteins and release the RNA. Following this step, quantitative reverse transcription PCR (qRT-PCR) was performed to analyze the RNA samples and assess the interactions between RNA and proteins in the complexes.

### Statistical analysis

All data were presented as mean ± standard deviation and analyzed using SPSS 22.0 (IBM, NY, USA) and GraphPad Prism 7.0 (GraphPad Software, La Jolla, CA). To determine significant differences between the two groups, Student’s *t*-test was employed, while ANOVA was used for assessing differences across multiple groups. Statistical significance was considered at a threshold of *p* < 0.05, indicating that observed differences were unlikely due to random chance.

## Results

### circPWWP2A was significantly induced both in UUO-induced kidney tissue and TGF-β-induced BUMPT cells

**Figure 1.**
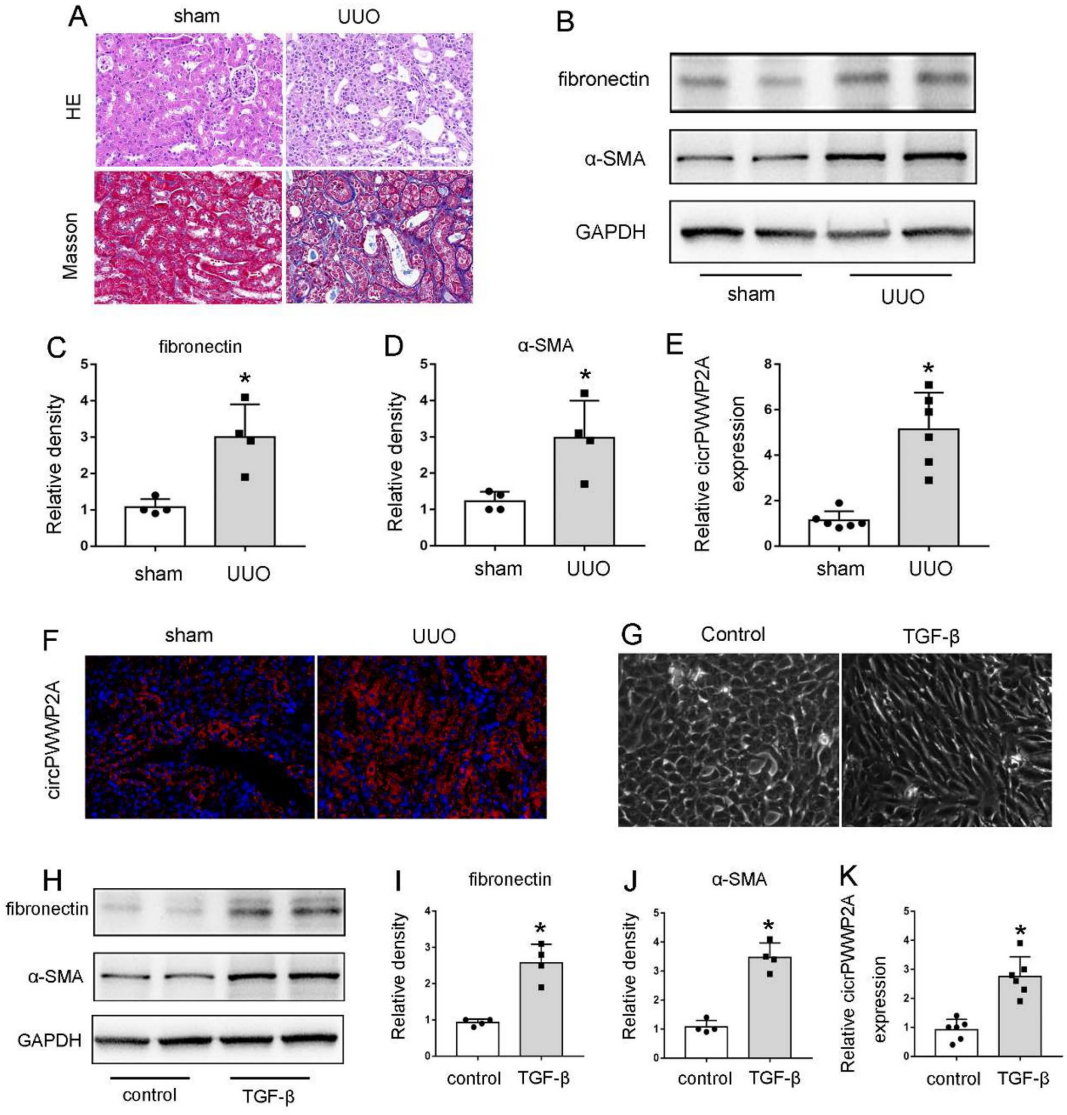
circPWWP2A Was highly expressed both in UUO-induced kidney tissue and TGF-β-induced BUMPT cells. (A–F) C57BL/6 mice underwent either UUO surgery or a sham procedure. Seven days post-surgery, the mice were euthanized, and the obstructed kidneys were collected for morphological and biochemical assessments. (A) Representative images of HE and masson staining at 400× magnification; (B) Western blot images showing FN (fibronectin), α-SMA (α-smooth muscle actin), and GAPDH (loading control); (C–D) densitometric analysis of FN and α-SMA, with the ratio of FN/GAPDH or α-SMA/GAPDH in sham controls set to 1.0, and other groups normalized against this control. Data are presented as mean ± SD (*n* = 4). *p < 0.05 (versus sham group); (E) qPCR analysis of circPWWP2A in mouse kidneys, with circPWWP2A levels normalized to GAPDH, and the ratio in control mice set to 1. Data are shown as mean ± SD (*n* = 6). *p < 0.05 (versus sham group); (F) In situ hybridization demonstrating increased circPWWP2A in kidney tissues at 400× magnification. (G–K) BUMPT cells were either untreated (control) or exposed to 5 ng/mL TGF-β for 48 h. Post-treatment, cell morphology was recorded, and lysates were collected for immunoblot analysis. (G) Cell morphology images under light microscopy at 400×; (H) Western blot images of FN, α-SMA, and GAPDH; (I–J) densitometric analysis of FN and α-SMA, with the ratio of FN/GAPDH or α-SMA/GAPDH in control cells set to 1.0, and other groups normalized accordingly. Data are presented as mean ± SD (*n* = 4). *p < 0.05 (versus control group); (K) qPCR analysis of circPWWP2A in BUMPT cells, with circPWWP2A levels normalized to GAPDH and the ratio in control cells set to 1. Data are shown as mean ± SD (*n* = 6). *p < 0.05 (versus control group).

To investigate the development of renal interstitial fibrosis, we began by utilizing a mouse model subjected to unilateral ureteral obstruction (UUO). Following a 7-day period of UUO treatment, the mice were euthanized, and the affected kidneys were harvested for detailed morphological and biochemical assessments. Initially, we performed hematoxylin and eosin (H&E) staining along with Masson trichrome staining to evaluate kidney injury and collagen deposition within the obstructed tissues. As illustrated in Figure 1A, UUO treatment resulted in significant tubular damage and pronounced collagen accumulation, as indicated by the increased blue staining in the Masson trichrome images, compared to the sham control kidneys. Further analysis involved measuring the levels of fibrosis-associated proteins, such as fibronectin (FN) and α-smooth muscle actin (α-SMA). Immunoblotting results revealed a substantial increase in both FN and α-SMA in the UUO-treated kidney tissues (Figure 1B), which was corroborated by semi-quantitative densitometric analysis (Figure 1C and D). These findings confirmed the successful establishment of the UUO mouse model. Additionally, real-time PCR analysis showed an upregulation of circPWWP2A in the UUO-treated kidneys relative to the controls (Figure 1E). In situ hybridization further localized the increase of circPWWP2A primarily to the renal tubules (Figure 1F). To further explore the role of circPWWP2A in renal fibrosis, we developed an in vitro model of renal tubulointerstitial fibrosis using TGF-β treatment. TGF-β is a critical mediator of renal interstitial fibrosis, affecting various cell types, including renal proximal tubules [20]. Consequently, TGF-β treatment of renal tubular cells serves as a widely accepted in vitro model for studying tubulointerstitial fibrosis. As shown in Figure 1G, untreated BUMPT cells exhibited a typical cobblestone morphology with robust cell-cell connections. In contrast, TGF-β treatment induced a spindle-shaped morphology in these cells, accompanied by a notable reduction in intercellular connections. Further, compared with control cell, our immunoblot results showed both FN and α‐SMA accumulated dramatically in the TGF‐β-treated cell (Figure 1H–J). Similar to in vivo model, our qPCR analysis indicated that the circPWWP2A was also induced in TGF‐β-treated BUMPT cells (Figure 1K). Taking together, these results indicated that circPWWP2A was highly expressed both in UUO-induced kidney tissue and TGF-β-induced BUMPT cells.

### Knockdown of circPWWP2A significantly inhibited TGF-β-induced BUMPT cell fibrosis, ROS, and mitochondrial dysfunction

**Figure 2.**
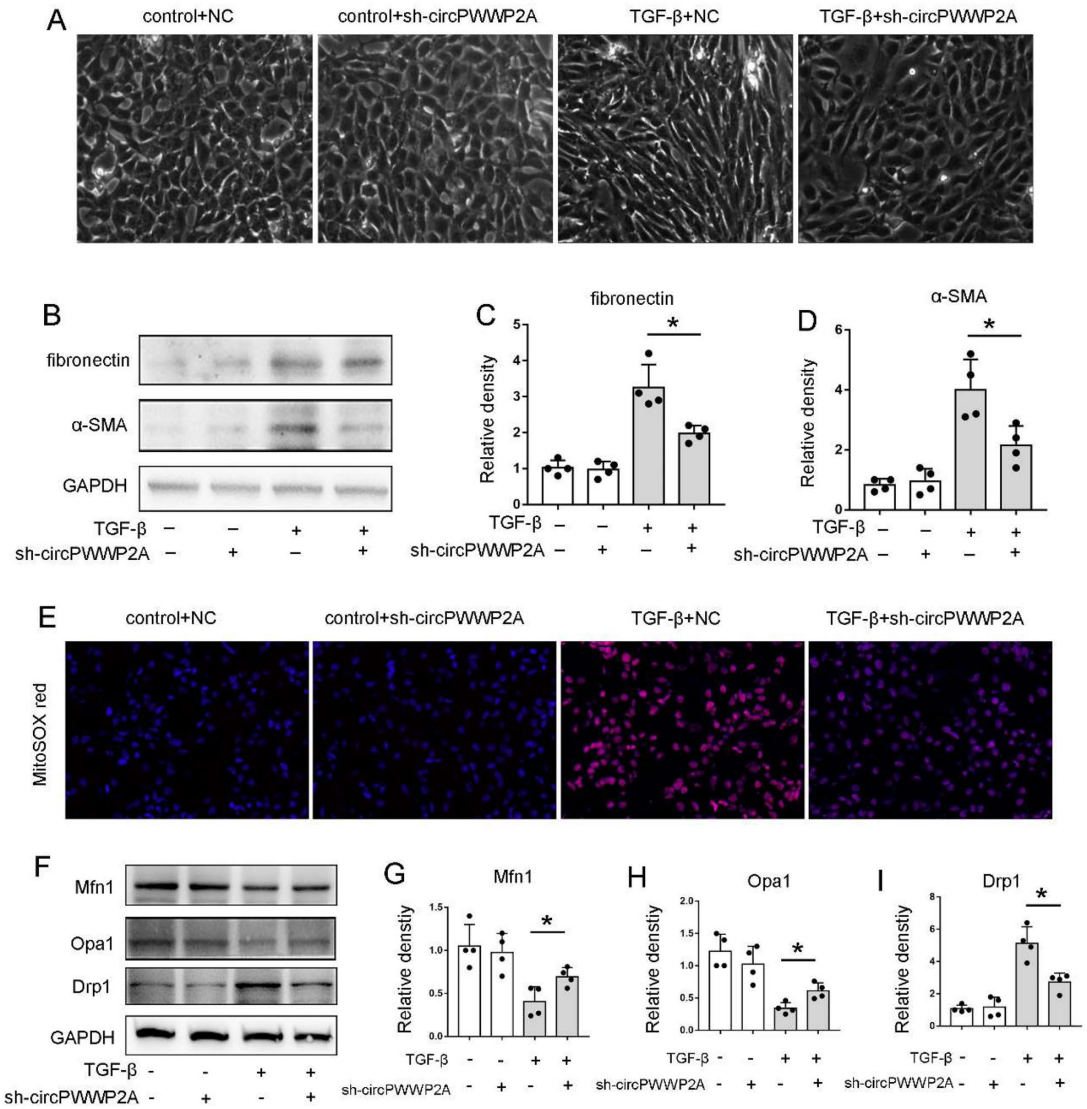
Knockdown of circPWWP2A significantly inhibited TGF-β-induced BUMPT cell fibrosis, ROS, and mitochondrial dysfunction. To explore the role of circPWWP2A in pathology, we created a circPWWP2A knockdown model using sh-circRNA. Cells were either left untreated (control) or exposed to 5 ng/mL TGF-β for 48 h. Following treatment, cell morphology was documented, and lysates were collected for Western blot analysis. (A) Light microscope images of cell morphology at 400× magnification; (B) Western blot images for FN, α-SMA, and GAPDH; (C–D) densitometric analysis of FN and α-SMA. The FN/GAPDH and α-SMA/GAPDH ratios for the (control + NC) group were set to 1.0, with other groups normalized to this control to calculate fold changes. Data are shown as mean ± SD (*n* = 4). *p < 0.05; (E) MitoSOX Red staining images at 400× magnification; (F) Western blot images for Mfn1, Opa1, Drp1, and GAPDH; (G–I) Densitometric analysis of Mfn1, Opa1, and Drp1. The Mfn1/GAPDH, Opa1/GAPDH, and Drp1/GAPDH ratios for the (control + NC) group were set to 1.0, with other groups normalized accordingly to determine fold changes. Data are presented as mean ± SD (*n* = 4). *p < 0.05.

To investigate the pathologic role of circPWWP2A, we established the circPWWP2A knockdown system by using sh-circRNA. Firstly, we confirmed the knockdown system was successfully established (data not shown). In contrast to the control cells, TGF-β-treated cells exhibited a spindle-shaped morphology with reduced intercellular connections. The addition of Sh-circPWWP2A alongside TGF-β partially mitigated these morphological alterations (Figure 2A). Western blot analysis was then performed to assess the expression levels of fibronectin (FN) and α-smooth muscle actin (α-SMA) in the BUMPT cells treated with TGF-β. Importantly, the changes of this two fibrosis protein markers during TGF‐β treatment were largely attenuated in sh-circPWWP2A cells (Figure 2B–D). These results indicated that blocking circPWWP2A could inhibit cell fibrosis. Then, we further measured the production of mitochondrial reactive oxygen species (mtROS) in BUMPT cells during TGF-β treatment by MitoSOX Red staining. Consistently, knockdown of circPWWP2A also significantly inhibited the ROS production following TGF-β treatment (Figure 2E). It has been indicated that mitochondrial dysfunction played an important role in renal fibrosis [21, 22]. Thus, we then detected the expression of mitochondrial division related gene Drp1 and mitochondrial fusion related gene Mfn1 and Opa1 by Western blot. Just as shown in Figure 2F–L, Knockdown of circPWWP2A significantly inhibited the up-regulation of mitochondrial division related genes Drp1 and the down-regulation of mitochondrial fusion related genes Opa1 and Mfn1 expression in TGF-β1-induced BUMPT cells, suggesting that circPWWP2A could promote mitochondrial dysfunction in renal fibrosis.

### Overexpression of circPWWP2A significantly promoted TGF-β1-induced BUMPT cell fibrosis, ROS, and mitochondrial dysfunction

**Figure 3.**
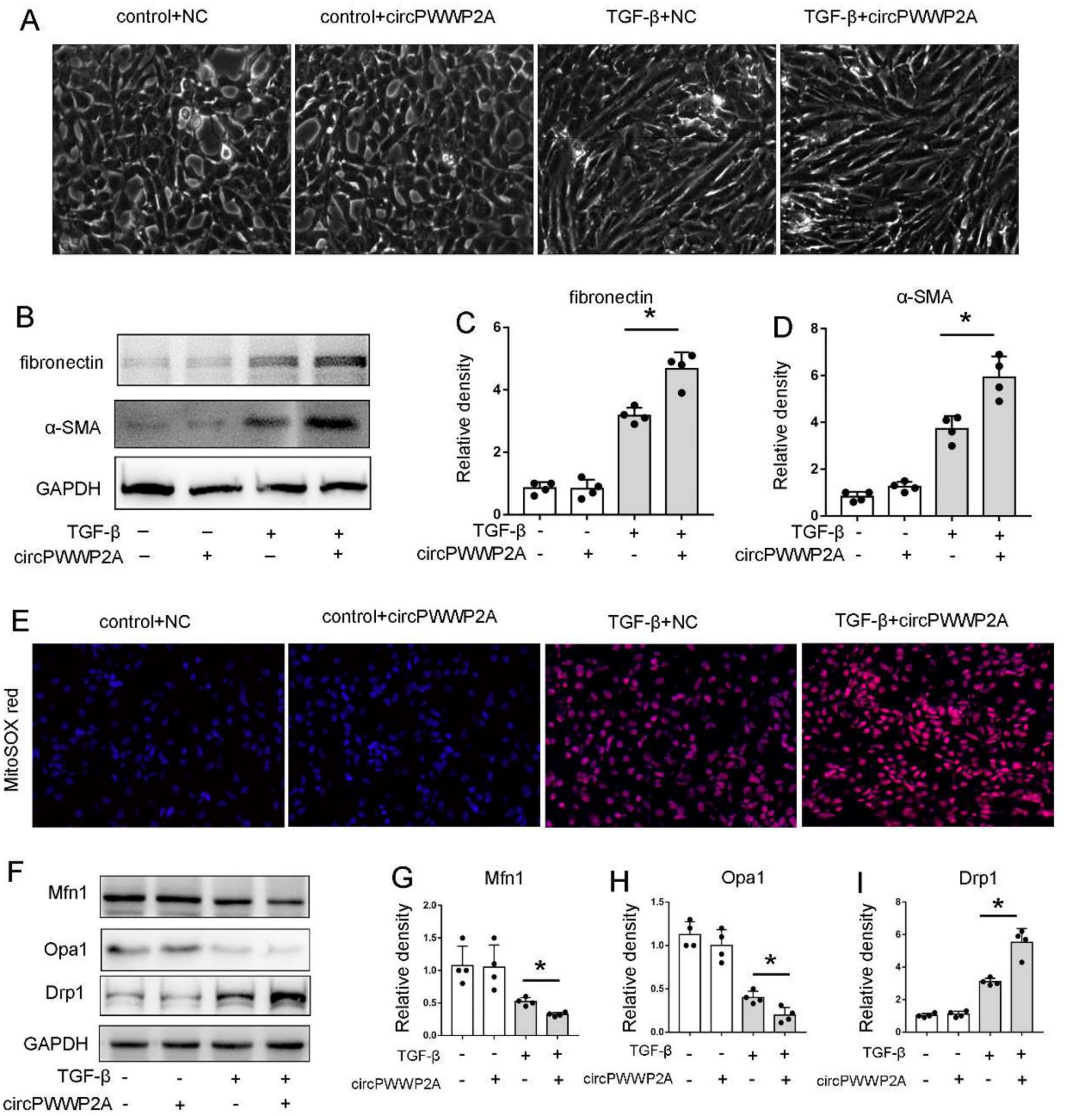
Overexpression of circPWWP2A significantly promoted TGF-β1-induced BUMPT cell fibrosis, ROS, and mitochondrial dysfunction. To explore the pathological impact of circPWWP2A, we developed an overexpression model using Lv-circRNA. Cells were either left untreated (control) or exposed to 5 ng/mL TGF-β for 48 h. Post-treatment, cell morphology was documented, and protein lysates were analyzed *via* Western blot. (A) Light microscope images of cell morphology at 400× magnification; (B) Western blot images for FN, α-SMA, and GAPDH; (C–D) Quantitative analysis of FN and α-SMA. The FN/GAPDH and α-SMA/GAPDH ratios for the (control + NC) group were assigned a value of 1.0, and other groups were compared to this control to calculate fold changes. Data are presented as mean ± SD (*n* = 4). *p < 0.05; (E) MitoSOX Red staining images at 400× magnification; (F) Western blot images for Mfn1, Opa1, Drp1, and GAPDH; (G–I) Quantitative analysis of Mfn1, Opa1, and Drp1. The Mfn1/GAPDH, Opa1/GAPDH, and Drp1/GAPDH ratios for the (control + NC) group were set to 1.0, with other groups normalized to these controls for fold change calculation. Data are shown as mean ± SD (*n* = 4). *p < 0.05.

To further investigate the pathologic role of circPWWP2A, we also established the circPWWP2A overexpression system by using Lv-circRNA. Firstly, we also confirmed the overexpression system was successfully established (data not shown). In contrast to sh-circPWWP2A, Lv-circPWWP2A enhanced the morphological changes (spindle‐shaped morphology with intercellular connection diminished) following TGF-β treatment (Figure 3A). The immunoblot analysis also indicated that the expression of FN and α‐SMA during TGF-β treatment were enhanced in Lv-circRNA cells (Figure 3B–D). Consistently, circPWWP2A promote ROS production in this model, as shown by MITOSOX RED staining (Figure 3E). Moreover, circPWWP2A also promotes the expression of mitochondrial division related genes Drp1 and inhibits the mitochondrial fusion-related genes Opa1 and Mfn1 in TGF-β treatment (Figure 3F–L). Taking together, these results further supported the conclusion that circPWWP2A could promote renal fibrosis, ROS production, and mitochondrial dysfunction.

### The expression of circPWWP2A showed an inverse relationship with the levels of miR-182

**Figure 4.**
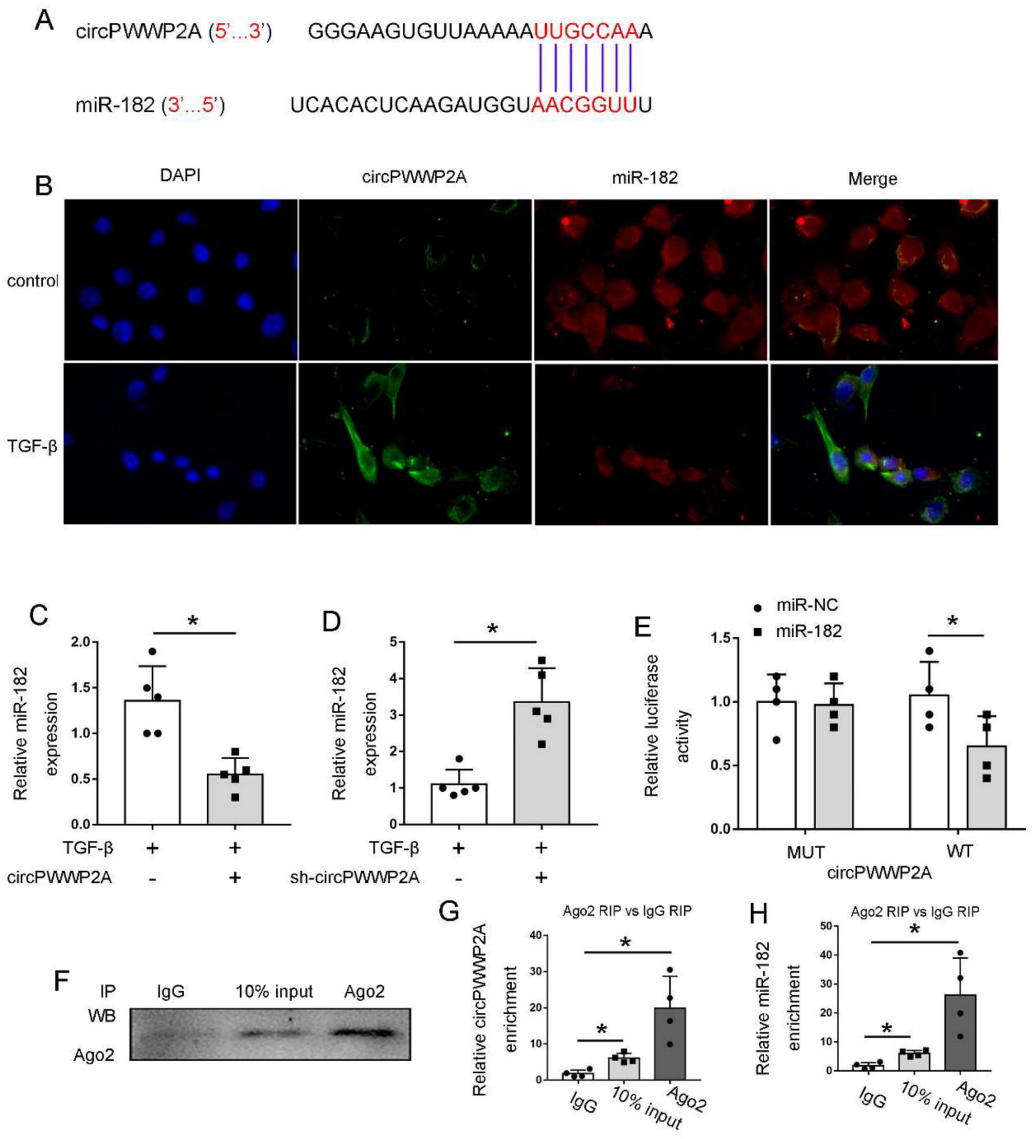
The expression of circPWWP2A was negatively correlated with the expression of miR-182. (A) Potential interaction site between circPWWP2A and miR-182; (B) FISH analysis showing the localization of circPWWP2A and miR-182 at 630× magnification. Cells were either left untreated (control) or exposed to 5 ng/mL TGF-β for 48 h before collection for analysis; (C) Expression levels of miR-182 in cells overexpressing circPWWP2A after TGF-β treatment. Results are shown as mean ± SD (*n* = 5). *p < 0.05; (D) miR-182 expression in cells with circPWWP2A knockdown following TGF-β treatment. Results are shown as mean ± SD (*n* = 5). *p < 0.05; (E) Dual-luciferase reporter assay confirming the interaction site between circPWWP2A and miR-182. Data are presented as mean ± SD (*n* = 4). *p < 0.05; (F) RIP assay demonstrating the interaction between circPWWP2A and miR-182; (G–H) Quantitative analysis of circPWWP2A and miR-182 enrichment from the RIP assay. Results are shown as mean ± SD (*n* = 4). *p < 0.05.

To investigate the molecular interactions of circPWWP2A, we employed bioinformatics tools to identify potential miRNA targets. Utilizing the Circular RNA Interactome database (https://circinteractome.nia.nih.gov/), we predicted miR-182 as a likely target of circPWWP2A (Figure 4A). To confirm these findings, we conducted fluorescence in situ hybridization (FISH) to examine the cellular localization of both circPWWP2A and miR-182, revealing their predominant presence in the cytoplasm. Importantly, Figure 4B demonstrates a negative correlation between the expression levels of circPWWP2A and miR-182. Further experiments involving the transfection of Lv-circRNA or sh-circRNA showed a corresponding decrease in miR-182 levels (Figure 4C and D), supporting the hypothesis that miR-182 might be a target of circPWWP2A. This hypothesis was validated through dual-luciferase reporter assays and RNA immunoprecipitation (RIP) assays. In the luciferase assays, plasmids with circPWWP2A wild-type (WT) or mutated (MUT) binding sites were co-transfected with either miR-NC or miR-182 mimics. The results indicated that the luciferase activity in the circPWWP2A WT with miR-182 mimic was significantly lower compared to other groups (Figure 4E), suggesting direct targeting by miR-182. Additionally, RIP assays showed that circPWWP2A was preferentially enriched in Ago2-containing miRNA ribonucleoprotein complexes compared to control IgG (Figure 4F–H). Overall, these findings confirm that circPWWP2A negatively regulates miR-182.

### ROCK1 is the target of miR-182

**Figure 5.**
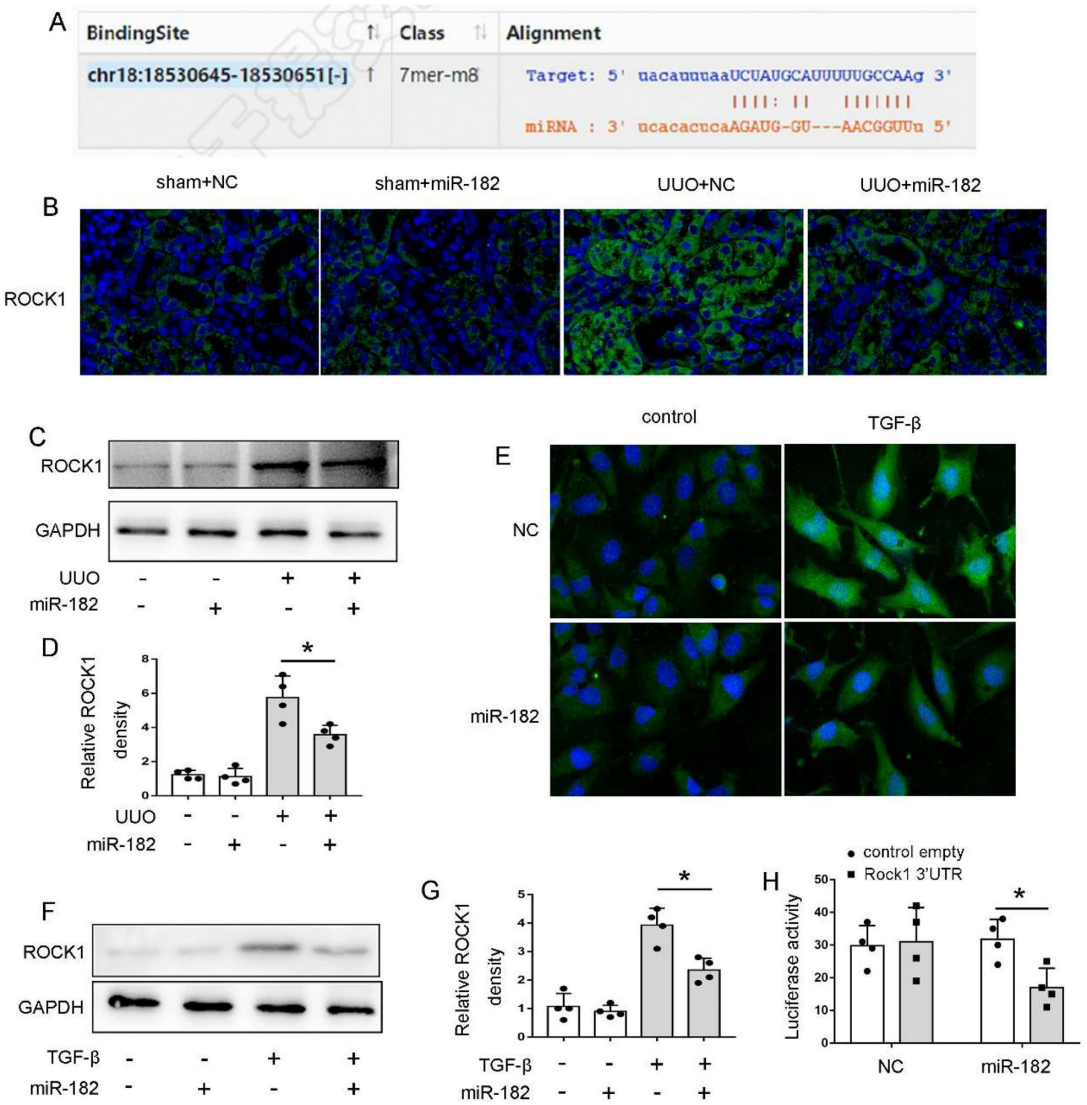
ROCK1 Is the target of miR-182. (A) Conserved binding site of miR-182 within the 3′-UTR of ROCK1 mRNA; (B) Immunofluorescence microscopy revealing the inhibition of ROCK1 expression by miR-182, 630× magnification. Mice received daily tail vein injections of either miR-182 mimic (3 mg/kg) or a control oligonucleotide LNA following UUO surgery. The obstructed kidneys were collected for analysis seven days post-surgery; (C) Western blot analysis demonstrating miR-182's suppression of ROCK1 expression. Mice underwent the same treatment as in (B), and kidney tissues were analyzed using immunoblotting with GAPDH as a loading control; (D) Quantitative analysis of ROCK1 band intensities from the Western blot; (E) Immunofluorescence images showing the effect of miR-182 on ROCK1 expression, 630× magnification. BUMPT cells were transfected with 200 nM miR-182 mimic or control for 24 h and then exposed to or untreated with TGF-β (5 ng/mL) for 48 h; (F) Western blot results illustrating the impact of miR-182 on ROCK1 expression in BUMPT cells, with GAPDH as a loading control; (G) Quantification of ROCK1 band intensities from the Western blot analysis; (H) Reporter assay assessing the interaction between miR-182 and ROCK1 3′-UTR. The suspected miR-182 binding site in the ROCK1 3′-UTR was inserted into the pMIR-REPORT vector and co-transfected with miR-182 mimic or control oligonucleotides to measure luciferase activity. miR-182 mimic specifically reduced luciferase activity associated with the ROCK1 3′-UTR vector. All data are presented as mean ± SD (*n* = 4), with *p < 0.05.

To explore how miR-182 influences renal fibrosis, we focused on its downstream target genes. Using bioinformatics tools from the Starbase database (http://starbase.sysu.edu.cn/index.php), we discovered a potential miR-182 binding site within the 3′-UTR of ROCK1 mRNA. This sequence is notably conserved across various species, as depicted in Figure 5A. In order to determine whether ROCK1 is indeed a miR-182 target, we initially assessed the impact of miR-182 on ROCK1 expression. As shown in Figure 5B, Immunofluorescence staining revealed a ROCK1 upregulation in murine renal tubule cells in UUO, and the miR-182 in vivo transfection significantly inhibited its expression. In addition, the immunoblot analysis further confirmed the in vivo transfection with miR-182 mimic led to decreased ROCK1 expression in UUO mice (Figure 5C and D). Similarly, in vitro experiments demonstrated that TGF-β treatment increased ROCK1 levels in BUMPT cells. This effect was significantly reduced when miR-182 mimics were introduced (Figure 5E). The inhibitory impact of miR-182 on ROCK1 expression was further confirmed through immunoblot analysis (Figure 5F and G). To ascertain the direct targeting of ROCK1 by miR-182, we conducted a luciferase microRNA target reporter assay. The 3′-UTR sequence of ROCK1 was inserted into the microRNA Luciferase reporter construct, which was subsequently co-transfected with either miR-182 mimic or NC oligonucleotides into HEK293 cells. As depicted in Figure 5H, co-transfection of miR-182 mimic (but not NC oligonucleotides) led to the inhibition of luciferase expression originating from the reporter construct containing ROCK1 3′-UTR (Figure 5H). Collectively, these findings suggest that miR-182 might directly target ROCK1 mRNA to suppress its expression in renal fibrosis.

### Blocking ROCK1 significantly inhibited TGF-β1-induced BUMPT cell fibrosis, ROS, and mitochondrial dysfunction

**Figure 6.**
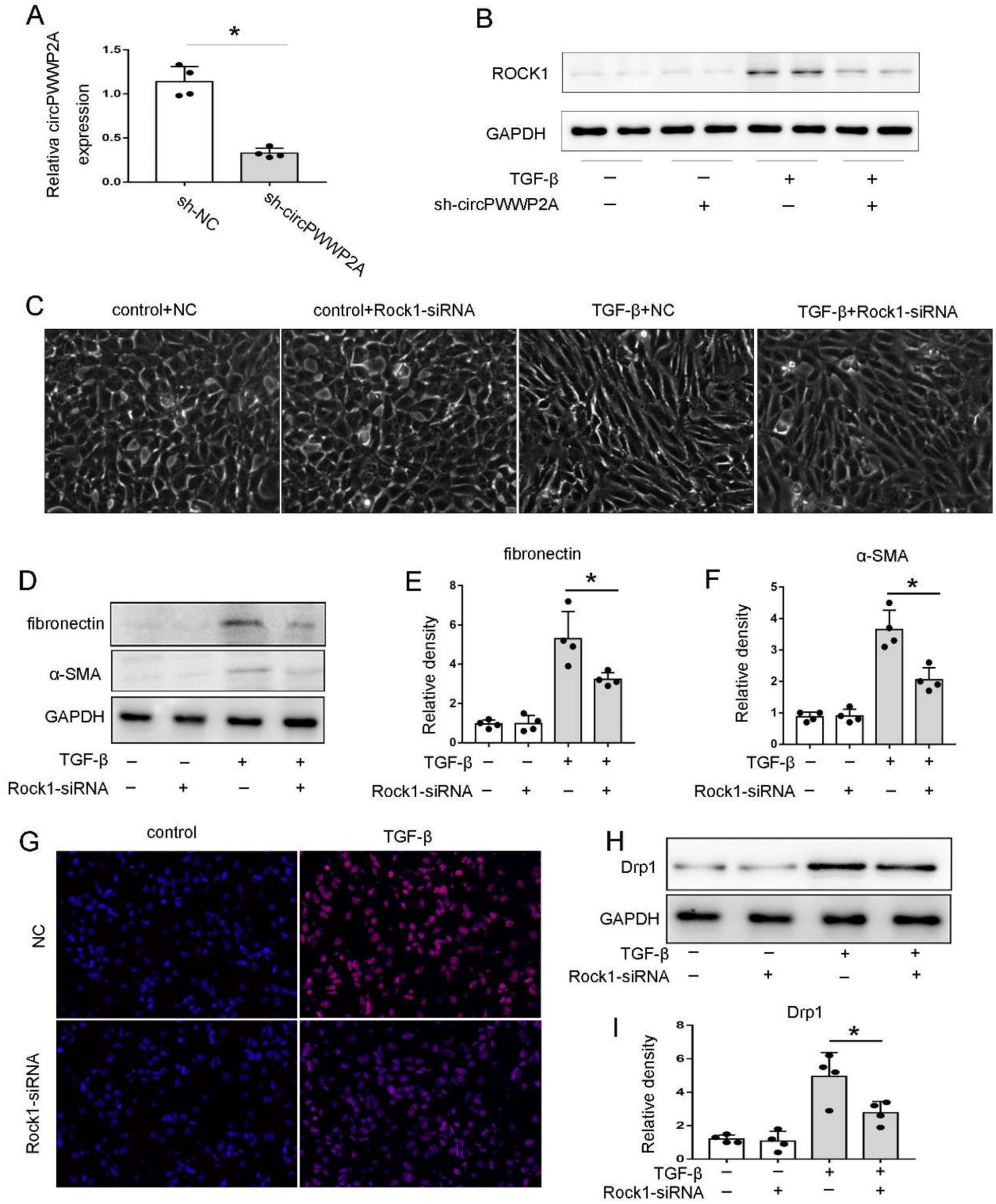
Blocking ROCK1 significantly inhibited TGF-β1-induced BUMPT cell fibrosis, ROS, and mitochondrial dysfunction. (A–B) To explore the role of circPWWP2A in renal interstitial fibrosis, we created a knockdown model using sh-circRNA. Cells were either untreated (control) or exposed to 5 ng/ml TGF-β for 48 h. (A) Quantitative PCR was used to measure circPWWP2A levels in BUMPT cells, with data normalized to GAPDH (internal control) and expressed relative to sh-NC cells, which were set as 1. Results are presented as mean ± SD (*n* = 4), with *p < 0.05 compared to sh-NC group; (B) Western blot images showing ROCK1 expression levels. (C–I) BUMPT cells were transfected with 40 nM ROCK1 siRNA or control oligonucleotides, then treated with or without TGF-β (5 ng/ml) for 48 h. (C) Light microscopy images of cell morphology, 400× magnification; (D) Western blot images for FN, α-SMA, and GAPDH; (E–F) densitometric analysis of FN and α-SMA. The ratios of FN/GAPDH or α-SMA/GAPDH in (control + NC) cells were set as 1, and other group ratios were compared to this baseline. Data are shown as mean ± SD (*n* = 4), with *p < 0.05; (G) MitoSOX red staining images, 400× magnification; (H) Western blot images for Drp1 and GAPDH; (I) Densitometric analysis of Drp1. The ratio of Drp1/GAPDH in (control + NC) cells was set as 1, and the ratios of other groups were normalized to this baseline. Data are expressed as mean ± SD (*n* = 4), with *p < 0.05.

To investigate the role of circPWWP2A in renal interstitial fibrosis, we established the circPWWP2A knockdown system by using sh-circRNA. The results in Figure 6A show that transfection with sh-circPWWP2A reduced the expression of circPWWP2A in BUMPT cells by approximately 70%, indicating successful transfection. Furthermore, knocking down circPWWP2A significantly suppressed the expression of ROCK1, suggesting that circPWWP2A positively regulating ROCK1 (Figure 6B). ROCK1 is widely involved in the regulation of somatic cell contraction, migration, skeleton remodeling, apoptosis and other processes, and plays a role in the occurrence and development of various diseases [23]. It has been reported that ROCK1 signaling pathway is associated with fibrotic lesions in various organs, such as pulmonary fibrosis, liver fibrosis, myocardial fibrosis, and renal fibrosis [24,25]. To assess the impact of ROCK1 on renal fibrosis, we investigated the effects of ROCK1 knockdown in BUMPT cells subjected to TGF-β treatment. As illustrated in Figure 6C, TGF-β treatment caused the cells to adopt a spindle-like shape with reduced intercellular connections. However, co-treatment with ROCK1-siRNA partially mitigated these morphological alterations. The immunoblot analysis also indicated that the expression of FN and α‐SMA during TGF-β treatment were attenuated in ROCK1-siRNA cells (Figure 6D–F). In addition, our MITOSOX RED staining indicated that blocking ROCK1 also could inhibit ROS production (Figure 6G). Importantly, our results also showed that ROCK1 knockdown could inhibit the expression of Drp1 (Figure 6H–L), indicating blocking ROCK1 could attenuated mitochondrial division during renal fibrosis.

### Inhibiting ROCK1 significantly inhibited renal fibrosis and mitochondrial dysfunction in UUO mice

**Figure 7.**
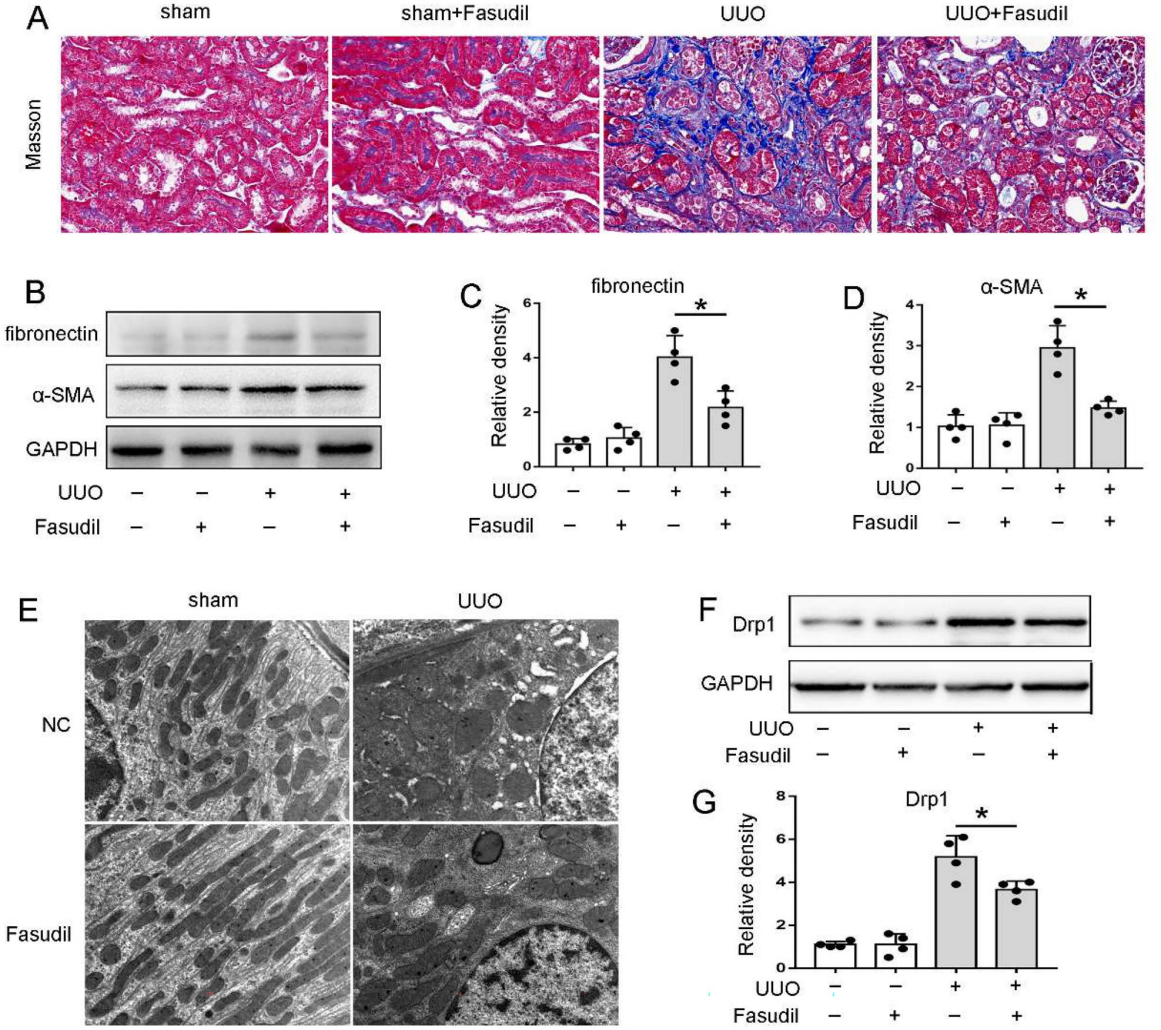
Inhibiting ROCK1 significantly inhibited renal fibrosis and mitochondrial dysfunction in UUO mice. C57BL/6 mice underwent either UUO surgery or a sham procedure. From the day of UUO surgery and continuing until euthanasia, the mice were intraperitoneally administered Fasudil (60 mg/kg), a known ROCK1 inhibitor. Seven days post-surgery, the mice were sacrificed to obtain obstructed kidneys for subsequent morphological and biochemical evaluations. (A) Masson staining images, 400× magnification; (B) Western blot analysis showing levels of FN, α-SMA, and GAPDH; (C-D) Quantitative analysis of FN and α-SMA. The FN/GAPDH and α-SMA/GAPDH ratios in (sham + NC) samples were set as 1, with other groups’ ratios normalized to this baseline. Data are shown as mean ± SD (*n* = 4), *p < 0.05; (E) Transmission electron microscopy (TEM) images of mitochondrial morphology in proximal tubular cells, 10,000× magnification; (F) Western blot analysis for Drp1 and GAPDH; (G) Quantitative analysis of Drp1. The Drp1/GAPDH ratio in (sham + NC) samples was set as 1, with other groups’ ratios adjusted accordingly. Data are expressed as mean ± SD (*n* = 4), *p < 0.05.

In order to further verify the role of ROCK1 in renal fibrosis, we further evaluated the effects of ROCK1 in vivo. Fasudil, a commonly used ROCK1 inhibitor, was used in this experiment. As depicted in Figure 7A, the UUO group exhibited an increased mesangial matrix, disrupted cellular organization, and extensive collagen fiber deposition in the interstitial area. However, administering Fasudil to UUO mice led to a reduction in the proportion of Masson staining-positive tissue. The immunoblot analysis also indicated that the expression of FN and α‐SMA in UUO mice were attenuated following Fasudil treatment (Figure 6B–D). Further, we examined mitochondrial morphology in kidneys by electron microscopy. Under normal conditions, mitochondria in renal tubular cells of sham + NC and sham + Fasudil mice were generally uniform in size and shape, predominantly displaying a tubular structure longer than 2 μm. In contrast, following UUO, the mitochondrial morphology in UUO + NC mice showed numerous small, rounded mitochondria with lengths less than 2 μm. This indicated fragmentation, swelling, matrix vacuolation, and cristae loss in the mitochondria (Figure 6E). Notably, tubular cell mitochondria in UUO + Fasudil group showed milder damage, indicating inhibiting ROCK1 could attenuate mitochondrial dysfunction. Moreover, our immunoblot analysis further showed that Fasudil could inhibit the up-regulation of mitochondrial division related genes Drp1 in UUO mice (Figure 7F–G). Taking together, these results indicate that blocking ROCK1 may attenuated renal fibrosis through inhibiting mitochondrial dysfunction.

## Discussion

Tubulointerstitial fibrosis represents a long-term and progressive condition impacting the kidneys, commonly seen with aging and CKD, regardless of the underlying cause. Both CKD and renal fibrosis impact approximately 50% of individuals over 70 years old and affect around 10% of the global population [4]. Despite ongoing research, the exact mechanisms underlying renal fibrosis are still not fully understood, and there are currently no effective treatments available. In the present study, we demonstrated a significant increase of circPWWP2A in renal tubular cell both in vivo and in vitro models of renal fibrosis. Functionally, inhibition of circPWWP2A could attenuate mitochondrial dysfunction and ROS production, finally inhibiting renal fibrosis. Whereas, supplementation of circPWWP2A led to more serve mitochondrial dysfunction, ROS production and renal fibrosis. Thus, circPWWP2A may act as a pro-fibrotic factor during renal interstitial fibrosis. Mechanistically, we further found the expression of circPWWP2A was negatively correlated with the expression of miR-182, and ROCK1 was the direct target of miR-182. It has been indicated that ROCK1 contributes to mitochondrial fission and oxidative damage in kidney diseases, including acute kidney injury (AKI) and CKD [26, 27]. Thus, our findings suggest that circPWWP2A may promote renal interstitial fibrosis by modulating miR-182/ROCK1-mediated mitochondrial dysfunction. This finding may provide a potential therapeutic target for renal fibrosis.

CircRNAs are a type of regulatory RNA that naturally occurs in cells, characterized by their single-stranded circular structure. It is widely found in cells and body fluids of different species. It is stable in structure, not easy to degrade, and has a high degree of conservation and tissue specificity [28]. In recent years, circRNAs have been shown to play critical regulatory role in renal fibrosis. For example, Wang et al. showed that circular RNA circBNC2 could inhibit epithelial cell G2-M arrest to prevent fibrotic maladaptive repair [29]. In another study, Cheng et al. showed that hsa_circ_0012138 had the potential to attenuate obstructive renal fibrosis [30]. In the present study, we demonstrated a significant increase of circPWWP2A in renal tubular cell both in vivo and in vitro models of renal fibrosis. Inhibition of circPWWP2A attenuated renal fibrosis, whereas supplementation of circPWWP2A led to more serve renal fibrosis. Thus, we have demonstrated compelling evidence to support a pro-fibrotic role of circPWWP2A in renal fibrosis. Importantly, Our FISH analysis revealed that circPWWP2A induction primarily occurs in the renal tubule cells within the renal cortex. Tubular damage is a key factor in the development of renal fibrosis [1]. Tubular epithelial cells are particularly affected by TGF-β1, which is secreted by both damaged tubules and infiltrating cells, triggering a fibrotic response that involves epithelial-to-mesenchymal transition (EMT) [1].

Next, we aimed to elucidate the molecular mechanism behind circPWWP2A's role in renal fibrosis. To achieve this, bioinformatics tools were employed to predict potential targets of circPWWP2A, revealing miR-182 as a key target. MicroRNAs (miRNAs) are small, non-coding RNA molecules, typically about 22 nucleotides long [31]. In mammalian cells, miRNAs control gene expression by attaching to the 3′-UTRs of target mRNAs, thereby preventing their translation. This mechanism is essential for regulating developmental processes, physiological functions, and the development of diseases such as renal fibrosis [31,32]. In the present study, our results indicated that the expression of circPWWP2A was negatively correlated with the expression of miR-182. And we further confirmed miR-182 was the direct target of circPWWP2A by dual-luciferase reporter assay and RIP assay. In published data, Sun et al. showed that miR-182 could inhibit kidney fibrosis by regulating TGF-β1/Smad3 pathway in autosomal dominant polycystic kidney disease [33]. In addition, Wang et al. found inhibition of miR-182-5p contributes to attenuation of lupus nephritis *via* Foxo1 signaling [34]. Moreover, miR-182 can inhibit hydrogen peroxide induced reduction of mitochondrial membrane potential and the release of Cytochrome c by targeting BNIP3 [33], indicating that miR-182 can regulate mitochondrial dysfunction and may play an important role in maintaining mitochondrial homeostasis.

To explore the role of miR-182 in renal fibrosis, our in vitro and in vivo investigations have pinpointed ROCK1 as a direct target of this microRNA. ROCK is the earliest identified Rho protein, including the highly homologous ROCK1 and ROCK2 subtypes. ROCK signaling pathway is an important signal transduction pathway in the body, widely involved in the regulation of somatic cell contraction, migration, skeleton remodeling, apoptosis and other processes, and plays a role in the occurrence and development of a variety of diseases [23]. It has been reported that ROCK signaling is associated with fibrotic lesions in various organs, such as pulmonary fibrosis, liver fibrosis, myocardial fibrosis, and renal fibrosis [25]. In UUO mice, ROCK was activated in renal fibrosis tissue, and inhibiting ROCK by Fasudil could alleviate UUO-induced renal interstitial fibrosis by inhibiting the expression of α-SMA, Collagen I and TGF-β/Smad signaling pathway [35]. Moreover, ROCK activation can promote Drp1-induced mitochondrial division, while blocking ROCK can inhibit the above mitochondrial division and the formation of smaller mitochondria, indicating that ROCK signaling is closely related to the maintenance of mitochondrial homeostasis [36]. In our present study, knockdown of ROCK1 both in vivo and in vitro inhibit renal fibrosis and mitochondrial dysfunction, suggesting ROCK1 not only served as an injurious role in mitochondrial homeostasis but also a pro-fibrotic factor in CKD.

In summary, our study provides the evidence of circPWWP2A was highly expressed in renal fibrosis tissues. Functionally, inhibition of circPWWP2A could attenuate mitochondrial dysfunction and ROS production, finally inhibiting renal fibrosis. Whereas, supplementation of circPWWP2A led to more serve mitochondrial dysfunction, ROS production and renal fibrosis. Mechanistically, circPWWP2A function as a ceRNA for miR-182 to modulate ROCK1 expression. ROCK1 could promote mitochondrial dysfunction, finally aggravating renal fibrosis. Thus, our findings suggest that circPWWP2A may promote renal interstitial fibrosis by modulating miR-182/ROCK1-mediated mitochondrial dysfunction, which may provide a potential therapeutic target for renal fibrosis.

## Data Availability

All data generated or analyzed during this study are included in this article. Further enquiries can be directed to the corresponding author.
